# A game theoretic approach to contract-based enviro-economic coordination of wood pellet supply chains for bioenergy production

**DOI:** 10.1186/s40807-023-00088-7

**Published:** 2023-11-21

**Authors:** Zahra Vazifeh, Fereshteh Mafakheri, Chunjiang An, Farid Bensebaa

**Affiliations:** 1 Department of Building, Civil, and Environmental Engineering, Concordia University, Montreal, QC Canada; 2 École Nationale d’administration Publique (ENAP), Université du Québec, Montreal, QC Canada; 3 Energy Mining and Environment (EM) Research Centre, National Research Council of Canada (NRC), Ottawa, ON Canada

**Keywords:** Bioenergy, Supply chain management, Carbon emission, Game theory, Bi-level programming, Coordination, Contracts, Communities, Sustainability

## Abstract

Wood pellets have gained global attention due to their economic availability and increasing demand for bioenergy as part of sustainable energy solutions. Management of the wood pellet supply chains, from feedstock harvesting to bioenergy conversion, is critical to ensure competitiveness in the energy markets. In this regard, wood pellets supply chain coordination can play a strategic role in enhancing the efficiency and reliability of bioenergy generation. This study proposes a contract-based coordination mechanism for wood pellet supply chains and compares its performance in alternative centralized and decentralized decision-making structures. A bi-level nonlinear game-theoretic approach with two economic and environmental objective functions is developed. It utilizes the concept of life cycle assessment in a Stackelberg leader–follower game to obtain the bioenergy equilibrium solutions. Further, this study examines the case of wood pellet supply chains in three remote Canadian communities. The aim is to showcase the practicality and significance of the proposed approach and interpret the findings. By focusing on these communities, the crucial role of supply chain coordination in fostering sustainable development, particularly, in the context of bioenergy generation is emphasized. The study colludes by advocating a number of avenues for future research.

## Introduction

The rising demand for bioenergy is driven by the global transition to renewable and sustainable energy sources for mitigating climate change and reducing dependence on fossil fuels. This has led to an increased interest in wood pellets as a prominent source for bioenergy (Duarah et al., 2022). Wood pellets, if sustainably harvested, could offer a renewable energy solution that can be sustained through responsible forestry practices. Moreover, wood pellets are recognized as a cleaner substitute for conventional fossil fuels, including coal, further emphasizing their environmental advantages. They have lower emissions of greenhouse gases and other pollutants when burned, making them more environmentally friendly. Wood pellets are a type of biomass fuel produced from compressed sawdust, wood chips, or other wood residues. With a moisture content below 10% and a bulk density of approximately 650 kg m-3 (Lee et al., 2020), wood pellets offer distinct advantages in terms of storage, handling, and overall practicality compared to alternative biomass forms. Furthermore, their uniform cylindrical shape has established them as a standardized, internationally traded commodity, with an estimated global market projection of 54 million tons by 2025 (Wolf et al., 2006).

The wood pellet supply chain primarily relies on forestry biomass as its main source of feedstock. This biomass is obtained through the harvesting of merchantable logs, which are then transported to sawmills for processing into lumber. Valuable co-products such as sawdust and wood chips are generated throughout this conversion process. Subsequently, these sawdust and wood chips are transported to pellet mills where they undergo pelletizing and classification procedures. The classification of wood pellets is determined by their properties and sources, adhering to the CAN/ISO-ISO 17225 solid biofuels standards (ISO 17225, 2021), with Grade A and Grade B representing the primary categories. Typically, Grade A wood pellets are utilized for residential or commercial heating purposes and are derived from mill residues and stem wood, whereas Grade B pellets are manufactured using a broader range of sources (NRC, 2016). Wood pellets find application in various contexts and can be efficiently combusted in different devices depending on the intended use. The combustion of wood pellets can be effectively modeled for two broad applications: residential pellet stoves and large-scale electricity generation.

The significant challenge of high production costs in wood pellet commercialization is multi-faceted. One of the core issues involves the need to ensure cost-effective transportation of feedstock from diverse and geographically distant sources to production facilities. Simultaneously, it is essential to account for the resulting carbon emissions generated throughout the supply chain. Adding to this complexity, uncertainties surrounding critical supply chain parameters, such as the seasonality of vital primary resources for wood pellet feedstock causes further intricacies (Mafakheri & Nasiri, 2014).

Pricing and logistics costs of biomass are not immune to the influence of international fluctuations in fossil fuel prices (Hamelinck et al., 2005). In addition, conflicts of interest among the diverse stakeholders involved in the supply chain introduce inconsistencies that hinder overall channel performance (Abusaq et al., 2022; Mafakheri et al., 2020). Consequently, to facilitate a swift transition towards a more environmentally sustainable fuel source, it is paramount to establish a well-coordinated and efficient wood pellet network design.

In pursuit of these objectives, a coordinated forestry-based wood pellet supply chain framework is introduced. This framework operates under a contract-based structure designed to encompass the hierarchical decision-making process. To address the intricacies of this challenge, a bi-level modeling approach is employed. Within this approach, two nested optimization problems interact, influencing each other's outcomes. The primary aim of this study is to propose an optimization model to minimize greenhouse gas (GHG) emissions across the entire wood pellet production life cycle while simultaneously maximizing profits for all stakeholders involved. This model is instrumental in reducing the environmental footprint associated with wood pellet production and ensuring optimal benefits for participants throughout the supply chain. To achieve this, a novel approach known as two-objective bi-level non-linear programming is employed. It is important to note that a multi-objective bi-level linear programming problem falls within the ambit of highly challenging and strongly nondeterministic polynomial-time hard (NP-hard) problems (Pakseresht et al., 2020; Zhang et al., 2023). Thus, specialized optimization techniques are essential for resolution. To overcome this computational hurdle, a transformation approach is adopted.

This study represents a pioneering effort, focusing on optimizing both economic and life cycle GHG emissions within the wood pellet supply chain, with a specific emphasis on non-cooperative stakeholders. Furthermore, the proposed optimization modeling framework amalgamates elements from the leader–follower game (Liu et al., 2021) and the life cycle optimization approach (Garcia-Velasquez et al., 2023), offering unique scope and strategic perspectives. To validate the framework, we conduct a case study in three off-grid communities situated in northern Canada. The results are subjected to thorough analysis and are further compared against a centralized model, offering comprehensive insights.

The organization of this paper is as follows: first, we provide essential background information and a concise overview of wood pellet supply chains, highlighting the challenges they entail. Next, in Sect. "Methodology", we present the formulation of our mathematical model and describe the dedicated solution algorithm employed. Sect. “Case study” introduces the details of the case study conducted, outlining its methodology and key parameters. Moving on to Sect. “Results and discussion”, we present the results and analysis derived from the case study. Finally, we conclude the paper by summarizing the main findings and suggesting potential avenues for future research.

## Background and literature review

Wood pellets have gained widespread popularity as an environmentally friendly energy source and have been extensively utilized in numerous countries (Erlich, 2009; Nunes et al., 2016; Proskurina et al., 2019). Consequently, scholars have directed their attention toward this sustainable energy solution, recognizing its significance and potential impact. In general, the existing literature can be classified into the following five sets, each addressing different aspects of wood pellets:

- Overview and challenges: various studies have reviewed the benefits, challenges, and future research directions in the wood pellet supply chain (Proskurina et al., 2017; Mohammadi, 2021). These works provide a comprehensive understanding of the overall landscape, identifying key obstacles and suggesting potential avenues for improvement.
- Production: researchers have examined the technological aspects and processes involved in the production and conversion of wood pellets (Di Marcello et al., 2017). These studies delve into the intricacies of the production methods, exploring the efficiency and effectiveness of different techniques.
- Market analysis and economics: understanding the market dynamics, pricing mechanisms, and economic viability of wood pellets is crucial for sustainable development. Several studies have focused on analyzing the market for wood pellets and evaluating their economic prospects (Peng et al., 2010). Such research aids in shaping effective strategies and policies to promote the growth of the wood pellet industry.
- Environmental impact analysis: assessing and mitigating the environmental impacts associated with wood pellet supply chains is of utmost importance. Researchers have conducted studies to evaluate the environmental consequences of wood pellet production and distribution (Myllyviita et al., 2012; Laschi et al., 2016). These works identify potential environmental risks and propose strategies for minimizing negative impacts.
- Policy and regulation: investigation of policy frameworks, regulations, and analysis of the impact of government policies on the development of wood pellet supply chains have been explored (Kittler, 2020). These studies shed light on the role of policy interventions in fostering the growth of the wood pellet industry while ensuring sustainable practices.

The review of literature shows that a limited number of studies have addressed the economic and environmental sustainability of pellet processing, as highlighted by Pergola et al. (2018), Wang et al. (2017), and Golonis et al. (2022). Despite the increasing significance of wood pellets as a renewable energy source, there is a noticeable dearth of comprehensive studies delving into the intricate interplay between economic viability and environmental impact throughout the wood pellet supply chain. Similarly, only a few investigations have ventured into the multifaceted challenges associated with the coordination of wood pellets from the supply side to conversion or consumer regions. This evident gap in the literature underscores the necessity for a more profound and holistic integration of the environmental and economic facets of wood pellet production and utilization.

Given the growing importance of wood pellets as a sustainable energy source, it is imperative to address these research gaps. This study seeks to bridge this void by exploring the synergies between environmental considerations and economic aspects within the context of wood pellet supply chain coordination. By doing so, a comprehensive understanding of the interplay between these two critical dimensions is achievable. This knowledge is not only vital for advancing the sustainability and efficiency of the wood pellet industry but also for shaping environmentally responsible energy policies and strategies that align with our overarching objectives of mitigating climate change and fostering economic development.

To evaluate the environmental performance of wood pellet production, the focus has been on life cycle GHG emissions (Gao & You, 2017). Roos and Ahlgren (2018) while considering the classification of life cycle assessment (LCA) into attributional and consequential approaches, conducted a comprehensive literature review on consequential LCA of bioenergy systems. The initial phase of the LCA process involves establishing the system boundaries, encompassing the upstream, midstream, and downstream aspects of the supply chain. Subsequent sections present a comprehensive overview of the wood pellet supply chain (Vazifeh et al., 2023a, 2023b), highlight existing gaps, and propose the implementation of game theoretic coordination tools as a viable solution.

### Wood pellet supply chains

The production process of wood pellets can be divided into three sections, namely upstream (feedstock supply), midstream (wood pellet production), and downstream (conversion), which are explained below to consider the life cycle of pellet production.

**Fig. 1:**
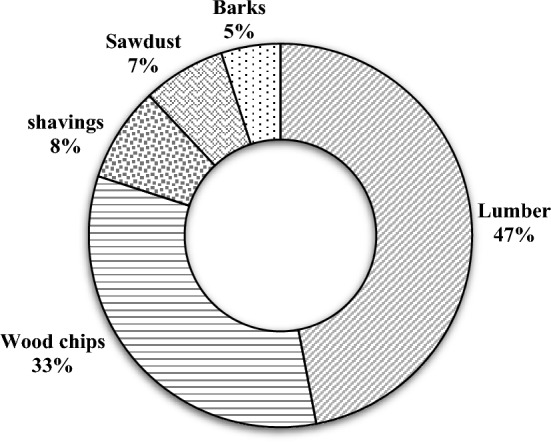
Proportion of forestry products and by-products

- Feedstock supply
  The wood pellet supply chain begins with the acquisition of raw materials, where the availability, quality, and cost of these materials play a crucial role in determining the feasibility and design of the supply chain (Lu & Rice, 2011). In general, forestry products and by-products can be classified into five categories, including lumber, wood chips, shavings, sawdust, and barks, as shown in Fig. 1 (Mobini, 2015). Currently, the primary raw material used in pellet production is sawdust, which is a by-product of the sawmill industry (Obernberger & Thek, 2010). Shavings and sawdust are highly preferred as raw materials due to their small particle size, low ash content, and low moisture content. Apart from sawmill residue, wood chips also serve as a residue directly obtained from the forestry process.
- Wood pellet production
  The wood pellet production process comprises several essential stages. Initially, raw materials such as sawdust, wood chips, or other wood residues are sourced from forestry operations, sawmills, or wood processing facilities. These raw materials undergo a meticulous processing phase to eliminate impurities like stones and metal particles. Subsequently, the processed wood material undergoes size reduction through grinding or chipping, ensuring the attainment of the desired particle size. This size reduction step is crucial for achieving uniformity and optimizing the combustion properties of the final pellets. To further enhance the quality of the pellets, the prepared wood particles are carefully dried to reduce moisture content. This drying process plays a pivotal role in improving energy efficiency and enhancing the combustion performance of the pellets. Once adequately dried, the wood particles are subjected to high-pressure compression, compacting them into dense and cylindrical pellets. This compression process often involves the utilization of a specialized pellet mill, where the wood particles are forcefully extruded through small holes in a die, resulting in the formation of pellets. In order to ensure the structural integrity of the pellets, heat may be applied during the compression process, activating the natural lignin present in the wood. This lignin acts as a binding agent, effectively holding the pellets together. Following the pelletization stage, the newly formed wood pellets are subjected to cooling and screening procedures to eliminate any fines or irregularly shaped pellets. Finally, the finished wood pellets are typically packaged in bags or bulk containers, ready for storage, transport, and distribution. It is important to note that the specific intricacies of the wood pellet production process can vary depending on factors such as the equipment used, desired pellet specifications, and the quality standards established by pellet manufacturers.
- Conversion

The conversion process of wood pellets is a crucial step in the production of bioenergy. The selection of a conversion technology determines the efficiency of the process and the quality of the end product. Two common conversion technologies are used for wood pellet conversion: gasification and direct combustion. Gasification technology has a higher capital cost but provides a higher energy efficiency rate, making it an attractive option for large-scale industrial applications. Direct combustion, on the other hand, has a lower capital cost but a lower energy efficiency rate, making it suitable for smaller-scale and more localized operations. The selection of a conversion technology should take into consideration the cost–benefit analysis, the desired level of efficiency, and the scale of the operation.

### Applications of game theory in the coordination of biomass supply chains

Applications of game theory have emerged as a valuable tool for analyzing and coordinating complex systems, including biomass supply chains. Game theory provides a mathematical framework for modeling strategic interactions among multiple stakeholders involved in the biomass supply chain, such as feedstock suppliers, biomass producers, processors, and end-users. By considering the behavior and decision-making of these stakeholders as strategic players, the game theory allows for a comprehensive analysis of their incentives, conflicts, and potential cooperation opportunities (Toktas-Palut, 2022). In the context of biomass supply chains, game theory can be applied to various aspects, including the following:

- Game-theoretic modeling of biomass supply chain coordination: Research by Vazifeh et al. (2021) proposed a game-theoretic model to analyze the coordination strategies among multiple biomass supply chain participants. The study considered factors such as pricing decisions, and quantity decisions aiming to optimize the overall supply chain performance and achieve coordination.
- Pricing and Contract Design: Gong et al. (2010) developed a game-theoretic framework to study contract design in a biomass supply chain. The research considered the interactions between the farmer (supplier) and the producer company and analyzed how contract designs affect supply chain coordination and efficiency.
- Cooperative Game Theory: Cooperative game theory has been employed to analyze cooperative behavior and coalition formations in biomass supply chains. Gao and You (2017) proposed a cooperative game model and investigate various profit allocation methods among biomass supply chain agents. The study concludes by recommending the nucleolus and equal profit methods as the most stable profit allocation methods.
- Resource Allocation and Optimization: Research has focused on using game theory to optimize resource allocation decisions in biomass supply chains. Tang et al. (2017) proposed a non-cooperative game-theoretic model for analyzing and identifying the best resource allocation strategy for the biomass industry owner.

The literature on the applications of game theory in the coordination of biomass supply chains demonstrates the effectiveness of this approach in addressing coordination challenges, pricing mechanisms, contract design, risk management, and resource allocation. These studies highlight the importance of strategic decision-making, cooperation, and efficient coordination among stakeholders to improve the overall performance and sustainability of biomass supply chains. Existing research in the field of game theory applied to biomass supply chain coordination has predominantly focused on simpler models, neglecting the incorporation of additional crucial factors. These factors include environmental considerations and the diverse economic preferences of individuals within the supply chain. By disregarding these significant elements simultaneously, the understanding of the inherent nature of real-world biomass supply chains remains limited. To overcome this limitation, this research emphasizes the development of more comprehensive and complex game-theoretic models that effectively capture the interplay between environmental concerns and the diverse economic motivations of stakeholders. Such advancements would contribute to a deeper understanding of the dynamics and complexities involved in coordinating biomass supply chains, facilitating the formulation of more realistic and effective strategies for sustainable biomass utilization. Motivated by this knowledge gap, the objective of this research is to develop a contract mechanism that effectively coordinates the wood pellet supply chain. By managing the interactions among participating agents, the aim is to steer their actions toward the benefit of the entire supply chain (Nugroho et al., 2022). In addition, a key focus of this research is to minimize GHG emissions throughout the life cycle of wood pellet production. By incorporating strategies to reduce GHG emissions, this study seeks to enhance the sustainability and environmental performance of the wood pellet supply chain. Through the design and implementation of this contract mechanism, the research aims to optimize both economic outcomes and environmental considerations, fostering a more sustainable and efficient biomass supply chain.

## Methodology

To systematically assess the performance of the contract coordination mechanism from the emission trading point of view, its whole life cycle needs to be investigated. LCA is employed to evaluate the GHG emissions of the proposed system. A comparative LCA is adopted to evaluate the carbon intensity of wood pellet production from hardwood forestry residues. Table 1 summarizes the three scenarios with corresponding decision-making (DM) structures and schematic views. In the first scenario, which is a centralized channel, the environmental impact (GHG) of the wood pellet production life cycle is calculated to provide a baseline. In scenario 2, net present value (NPV) and GHGs in the case of a decentralized decision-making structure are investigated, and scenario 3 is designed to show the impact of revenue-sharing and quantity discount contract coordination techniques.

**Table 1:** Scenarios definition

Scenario	DM structure	Schematic view
1	Centralized	
2	Decentralized	
3	Coordinated	

A case study of northern Canadian communities is considered to validate the proposed models. Due to the low economy of scale of bioenergy in these communities, direct combustion is selected as the conversion technology. The case study considers three Quebec northern communities in the Nunavik region. Although Canada has access to a great number of biomass resources from various sources, there is strictly no possibility of relying upon a local biomass supply in this region because the unsuitable vegetation texture of the region does not support any reliable sources of biomass. Therefore, wood pellets produced in three selected pellet mills must be imported to these communities. The mathematical modeling of the biomass supply chain problem is described in the following sections.

- Centralized scenario
  For a centralized scenario, the GHG of the wood pellet production life cycle is calculated to establish a baseline. Therefore, to estimate the GHG emissions for the production and conversion of 1 kg wood pellet, the open-source software, openLCA version 1.11 (openLCA, 2006) is used. This software is widely used for life cycle assessments and provides a comprehensive platform for data analysis and modeling. Data come from the Ecoinvent database (V3.6), which is a widely recognized and trusted source of life cycle inventory data. Furthermore, the consultation of peer-reviewed literature enhances the acquisition of additional data and valuable insights. To comprehensively assess the environmental impact, the Tool for the Reduction and Assessment of Chemical and Other Environmental Impacts (TRACI) methodology is utilized. TRACI considers a range of environmental factors, including energy consumption, greenhouse gas emissions, water usage, air pollution, and waste generation. By employing TRACI, a holistic evaluation of the environmental implications associated with the studied subject is achieved. For a specific reference, Table 2 provides an overview of the raw materials and energy requirements to produce 1 kg of dry wood pellets.
- Decentralized scenario

**Table 2:** Raw materials used to produce 1 kg wood pellet

Flow	Amount	Unit
Electricity, medium voltage	0.18	kWh
Heat, central or small-scale	9	MJ
Maize starch	0.005	kg
Packaging film (low-density polyethylene)	0.002	kg
Sawdust	0.57	kg
Hardwood shavings	0.3	kg
Water	3.00E−05	m3
Wood chips	0.13	kg

In the dynamic and fiercely competitive business environment of today, every participant within a supply chain functions as an autonomous entity, driven by the pursuit of maximizing their individual profits. Consequently, a decentralized decision-making system takes shape, where each member operates independently, often with conflicting interests. Recognizing this complex interplay of interests, the Stackelberg game emerges as a valuable and influential tool for modeling the behavior of supply chain participants. By employing the Stackelberg game, the intricate dynamics of the supply chain, characterized by varying power dynamics and conflicting objectives, can be effectively captured and analyzed. This game-theoretic approach enables a deeper understanding of the strategies and decision-making processes employed by different supply chain members, ultimately facilitating improved coordination and performance within the supply chain ecosystem. The Stackelberg game comprises two distinct decision-making levels: the leader(s) and the follower(s). The leader, representing the upper-level problem, assumes the role of decision-maker and takes the initial action, while the follower(s) subsequently make their decisions based on the leader's choice.

**Fig. 2:**
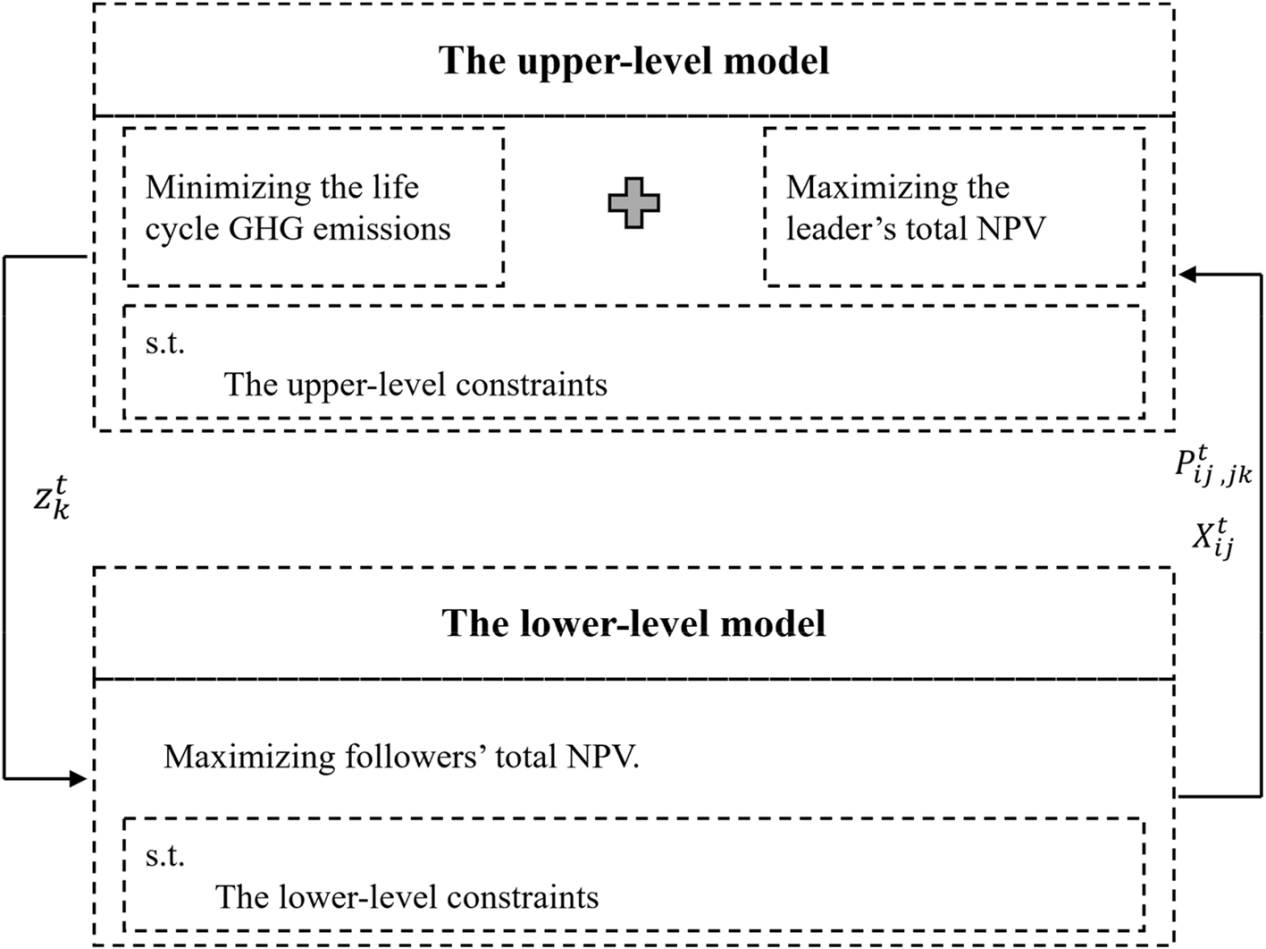
Structure of the proposed multi-objective bi-level model

In the context of this study, the upper-level problem encompasses two objective functions: economic and environmental considerations. The primary objective in the upper level involves maximizing the net present value (NPV) for the leader (as illustrated in Fig. 2). Adhering to the principles of the Stackelberg game, the leader possesses comprehensive information about the supply chain and holds the advantage of making decisions before others. A study by Vazifeh et al. (2021) examined power structures within the biomass supply chain of Canadian northern communities, and their findings revealed that if the conversion facilities assume the role of the leader in the supply chain, it leads to enhanced efficiency in terms of bioenergy generation and minimized side payments. Therefore, in this study, the downstream conversion facilities are designated as the leader. As the initiator and first mover in the pellet supply chain, the conversion facilities not only have the privilege of making decisions first to maximize their total NPV but also shoulder the responsibility of taking care of mitigating life cycle greenhouse gas (GHG) emissions throughout the entire supply chain. By adopting the Stackelberg game framework, this study recognizes the autonomous decision-making nature of supply chain members and employs it to examine the interplay between economic objectives, environmental considerations, and power dynamics within the biomass supply chain. Through the strategic positioning of the conversion facilities as the leader, this research aims to optimize both economic outcomes and environmental performance while effectively managing life cycle GHG emissions across the entire supply chain.

The leaders’ objectives (upper-level) encompass maximizing the communities' NPV and minimizing the life cycle GHG emissions associated with wood pellets, as outlined in Eqs. 1 and 2, respectively. It is worth nothing that the communities’ NPV is defined as the total profit that communities earn with replacing diesel with wood pellets as the main source of electricity generation. Equation (1) includes the cost of generating ${z}_{k}^{t}$ kwh from diesel (representing the savings that could be earned by switching to wood pellets) minus the total cost of generation ${z}_{k}^{t}$ from wood pellet and the cost of satisfying the reminder of demand ${({D}_{k}^{t}- z}_{k}^{t})$ with diesel. The decision variable of the upper-level problem is ${z}_{k}^{t}$, which is the kWh of electricity generation from biomass in conversion facility *k* at time *t*. ${z}_{k}^{t}$ is a function of${X}_{jk}^{t}$, the quantity of transported pellet to the location of conversion facility *k*:

1 $$\mathrm{Max} {NPV}_{C}= {\sum }_{k} \sum_{t} [({z}_{k}^{t} {LD}_{k })- {z}_{k}^{t} \cdot {f}_{c}^{-1} \left({P}_{jk}^{t}+ {C}_{con}\right)-{LD}_{k} {({D}_{k}^{t}- z}_{k}^{t})]$$

2 $${\mathrm{Min} F}_{LC}={CF}_{S}+{CF}_{P}$$

where $${CF}_{S}$$ is the carbon footprint of the upstream (supply) process including, harvesting$$({CF}_{ha})$$, debarking ($${CF}_{de}$$), sawing ($${CF}_{sa}$$), and transportation to the location of pellet factory ($${CF}_{tp})$$, Eq. (3). $${CF}_{P}$$ is the carbon footprint of the midstream (pellet production) process including drying, milling, pelletizing, cooling, and transportation to the location of conversion facilities, which are represented by $${CF}_{dr} , {CF}_{mi}$$,$${CF}_{pe}$$,$${CF}_{co}$$, and $${CF}_{tc},$$ respectively, in Eq. (4):

3 $${CF}_{S}=\sum_{i} \sum_{j}{{X}_{ij}^{t}}_{ }{(CF}_{ha}+{CF}_{de}+{CF}_{sa}+ {CF}_{tp (ij)})$$

4 $${CF}_{P}=\sum_{j} \sum_{k}{{X}_{jk}^{t}}_{ }({CF}_{dr}+{CF}_{mi}+{CF}_{pe}+{CF}_{co}+ {CF}_{tc(jk)})$$

Meanwhile, due to limited information, the supplier and pellet factory take actions after the converter and only tend to care about their own profit. Thus, after the realization of the leader’s decisions, the followers will react accordingly to optimize their own objective, which is maximizing followers’ total NPV (Eqs. 5, 6). The NPV for the pellet factories is calculated considering the revenue generated from selling pellets to communities minus the overall cost of pellet production. The latter comprises several components, namely ${C}_{dr}$(cost of drying), ${C}_{mi}$ (cost of milling), ${C}_{pe}$ (cost of pelletization), ${C}_{co}$ (cost of cooling), and ${C}_{tc}$ (cost of transportation to the communities' location). In a similar fashion, the NPV for the suppliers is determined considering the revenue obtained from selling feedstock to pellet factories minus the total cost of feedstock preparation. The latter encompasses several elements, including ${C}_{ha}$(cost of harvesting), ${C}_{tr}$ (cost of raw material's transportation for preprocessing), ${C}_{de}$ (cost of debarking), ${C}_{sa}$ (cost of head sawing), and ${C}_{tp}$ (cost of transportation to the pellet factories' location).

The decision variables of the lower-level problem are ${P}_{ij}^{t}$ (feedstock price from supplier *i* to pellet factory *j* at time *t*), ${P}_{jk}^{t}$ (wood pellet price transported from pellet factory *j* to *community k* at time *t*) pellet and ${X}_{ij}^{t}$ (the quantity of feedstock transported from supplier *i* to pellet factory *j* at time *t*). The price of wood pellets and the quantity of required biomass feedstock are decided by the pellet factory based on the quantity of wood pellets ordered by conversion facilities. Subsequently, suppliers make decisions regarding the price of feedstock:

5 $$\begin{aligned}\mathrm{Max} {NPV}_{P}&= {\sum }_{j} \{ \sum_{t} [(\sum_{k}{X}_{jk}^{t} {P}_{jk}^{t})\\&\quad- {X}_{ij}^{t} ({P}_{ij}^{t}+ {C}_{dr}+ {C}_{mi}+{C}_{pe}+{C}_{co}+{C}_{tc})]\}\end{aligned}$$

6 $$\begin{aligned}\mathrm{Max} {NPV}_{S}&= {\sum }_{i} \{ \sum_{t} [(\sum_{j}{X}_{ij}^{t} {P}_{ij}^{t})\\ &\quad- {\alpha }_{m}{X}_{ij}^{t} ({C}_{ha}+{C}_{de}+{C}_{sa}+{C}_{tp})]\}\end{aligned}$$

Constraints of the model are presented in Eqs. 7, 8, 9, 10, 11, 12, 13. Equation (7) demonstrates that at time ‘*t*’, the quantity of ordered feedstock cannot exceed the capacity of the suppliers. Similarly, Eq. (8) considers the capacity of wood pellet plants. Equation (9) indicates that the electricity generation in kWh from wood pellets cannot exceed the quantity of wood pellets purchased by the conversion facilities, multiplied by the conversion rate of wood pellets to electricity (${\mathrm{f}}_{\mathrm{c}})$. Equation (10) considers the electricity demand of the communities and the capacity of electricity generation plants. It ensures that the electricity generation in kWh does not exceed the lower value between the communities’ electricity demand and the capacity of the generation plants. Equations (11, 12, 13), ensure that the variables cannot have negative values in the solution space:

7 $$\sum_{j}{{X}_{ij}^{t}}_{ }\le {S}_{i}$$

8 $$\sum_{k}{{X}_{jk}^{t}}_{ }\le {S}_{j}$$

9 $$\sum_{k}{z}_{k}^{t}\le \sum_{k}{{X}_{jk}^{t}}{\mathrm{f}}_{\mathrm{c}}$$

10 $${z}_{k}^{t}\le \mathrm{min }( {D}_{k}^{t}, {f}_{c}^{-1}*{Z}_{k}*720)$$

11 $${X}_{ij}^{t}\ge 0$$

12 $${X}_{jk}^{t}\ge 0$$

13 $${z}_{k}^{t}\ge 0$$

- Coordinated scenario

In the coordinated scenario, a contract mechanism is designed using game theory to model the strategic interactions between different parties in the wood pellet supply chain. By considering the incentives and actions of all parties, contracts can help to align the interests of all parties and create a stable and efficient supply chain. In this study, revenue-sharing (Cachon & Lariviere, 2005) and quantity discount (Weng & Wong, 1993) contracts coordination mechanism are used to encourage collaboration between suppliers, pellet factories and conversion facilities. Revenue-sharing allows for profits to be shared between the two parties, creating an incentive for pellet producers to provide high-quality wood pellets and for conversion facilities to purchase it at a fair price. This helps to reduce transaction costs and promote long-term relationships between the parties involved. Quantity discount contracts, on the other hand incentivize customers to increase their order size or volume by providing cost savings as the quantity increases. By coordinating the volume and price of purchases, the parties involved can optimize their operations and improve their bottom line. Under these contracts, the conversion facility can obtain wood pellets from the pellet factory at a discounted price while as a compensation, the conversion facility must share his revenue with the pellet factory at a certain revenue-sharing rate, say *r* (0 ≤ *r* ≤ 1), where *r* represents the portion of the revenue to be shared with the pellet factory. The mathematical formulation of this model is presented below:

14 $$\begin{aligned} & \mathrm{Max} {NPV}_{C}=\left(1-r\right) {\sum }_{k,t} [{(z}_{k}^{t}\cdot {LD}_{k })\\ &\quad- {Z}_{k}^{t} {f}_{c}^{-1} \left({P}_{jk}^{t}+ {C}_{\mathrm{con}}\right)-{LD}_{k} {({D}_{j}^{t}- z}_{k}^{t})]\end{aligned}$$

where ${P}_{jk}^{t}$ is a function of the quantity of wood pellets shipped to the location of the conversion facility and is dependent on the portion of demand that is satisfied in the specific period:

15 $${P}_{jk}^{t}={P}_{j}^{u}-\left({P}_{j}^{u}- {P}_{j}^{l}\right) \frac{{{X}_{jk}^{t}}}{{D}_{j}^{t}}$$

If we define ${R}_{k}^{t}$ as the total revenue of the conversion facility *k* in time *t*, the objective function of the pellet factory is presented in Eq. (16):

16 $$\begin{aligned} & \mathrm{Max} {NPV}_{P}= {\sum }_{j,t} \sum_{k}[\left({R}_{k}^{t}.r\right)\cdot {(X}_{jk}^{t}\cdot {P}_{jk}^{t})]\\ &\quad- {X}_{ij}^{t} \cdot ({P}_{ij}^{t}+ {C}_{dr}+ {C}_{mi}+{C}_{pe}+{C}_{tc})\}\end{aligned}$$

We will examine another quantity discount contract between suppliers and pellet factory. Therefore ${P}_{ij}^{t}$ is defined as Eq. (17). To encourage the wood pellet factory to purchase more, the suppliers offer a quantity discount price on each order:

17 $${P}_{ij}^{t}={P}_{i}^{u}-\left({P}_{i}^{u}- {P}_{i}^{l}\right) \frac{{{X}_{ij}^{t}}}{{S}_{i}^{t}}$$

The objective function of suppliers and the constraints in this scenario remain unchanged (Eqs. 5, 6, 7, 8, 9, 10, 11, 12, 13).

## Case study

**Fig. 3:**
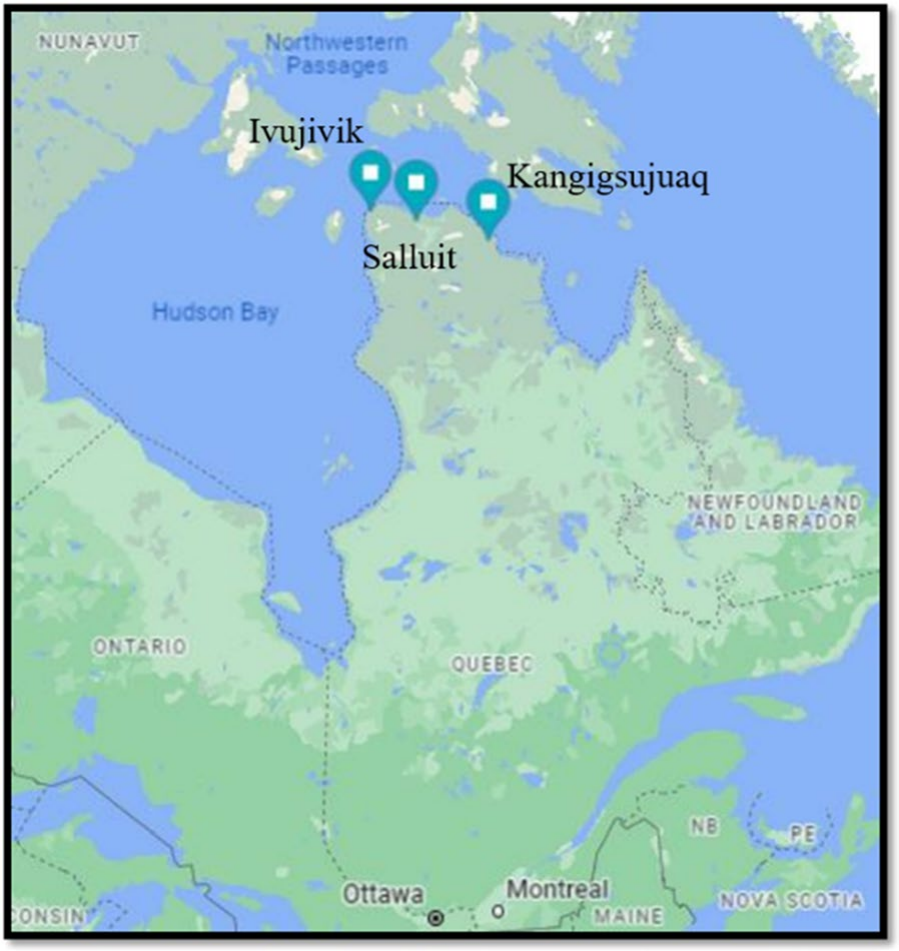
Location of the selected Communities

To assess the viability of the designed model, a comprehensive case study in three remote Canadian communities: Kangigsujuaq (KA), Salluit (SA), and Ivujivik (IV) is considered. These communities present a distinctive geographical context from the standpoint of supply chain dynamics, as their access is solely through water routes. Specifically, they can be reached either via the Hudson Bay from the east or the Labrador Sea from the west (as depicted in Fig. 3).

The purpose of this case study was to evaluate the efficacy of our proposed models in the context of these remote Canadian communities. By examining the adoption and utilization of wood pellets within the communities, we sought to determine the extent to which wood pellets can serve as a viable alternative for meeting their electricity and heat requirements. This analysis took into consideration various factors, such as the availability and accessibility of wood pellets, transportation logistics, community-specific energy demands, and environmental considerations.

By focusing on these unique locations and their distinct supply chain characteristics, our case study aimed to provide valuable insights into the feasibility and effectiveness of wood pellets as a sustainable energy solution for remote communities that rely on water routes for access. The findings of this study contribute to the broader understanding of the practical applications and potential benefits of wood pellets as an alternative energy source in similar remote settings. The parameters pertaining to the case study and their references are presented in Table 3.

**Table 3:** Parameters of the model and references

Definitions	Symbols and units	Value	References
Transportation cost from supplier ‘*i*’ to pellet factory ‘*j*’	$${T}_{ij}$$($/kg)	Appendix 1	(Vazifeh et al., 2021)
Capacity of supplier ‘*i*’	*S*_*i*_ (kg)	Appendix 2	(Mafakheri et al., 2020)
Biomass price of supplier ‘*i*’ with and without discount	$${P}_{i}^{l,u}$$($/kg)	Appendix 2	(Mafakheri et al., 2020)
Biomass harvesting cost for supplier ‘*i*’	$${\mathrm{hs}}_{i}$$($/kg)	0.4 (for all suppliers)	(Vazifeh et al., 2021)
Wood pellet ordering cost from pellet factory ‘*j*’ with and without discount	$${P}_{j}^{l,u}$$($/kg)	Appendix 3	(Mobini, 2015)
Conversion rate of wood pellet to electricity	$${fc}$$(kWh/kg)	4.7, 4.8, 4.6	(Mobini, 2015)
Loading factor of energy convertor ‘*k*’	$${Lf}_{k}$$(%)	0.80, 0.85, 0.80	(Vazifeh et al., 2023a, 2023b)
Electricity generation cost from biomass	$${LB}_{k}$$($/kWh)	0.046, 0.044, 0.048	(Vazifeh et al., 2021)
Electricity generation cost from diesel	$${LD}_{k}$$($/kWh)	0.208, 0.25, 0.207	(Mafakheri et al., 2020)
Demand in energy convertor ‘*k*’ at time t	$${D}_{k}^{t}$$(kWh)	Appendix 4	(Mafakheri et al., 2020)
Capacity of electricity generation	$${Z}_{k}$$(kW)	500 (for all communities)	(Mafakheri et al., 2020)

## Results and discussion

The proposed modeling framework in this paper results in a MOBNLP programming problem, which cannot be solved directly using any off-the-shelf global optimizers (Zhang et al., 2023). Therefore, the solution approach involves transforming the original problem into a single-level using Karush–Kuhn–Tucker (KKT) conditions. Kim and Ferris (2019) introduced an extended mathematical programming (EMP) approach, utilizing the Karush–Kuhn–Tucker (KKT) conditions, to reformulate the bi-level problem into the Mathematical Program with Equilibrium Constraints (MPEC) framework. This reformulated problem is solved using an MPEC solver within the General Algebraic Modeling System (GAMS) (GAMS, 2016). Their method demonstrated superior performance in terms of accuracy compared to traditional complementarity-based models, which necessitate manual computation of the Lagrangian derivatives.

In this study, the EMP tool in GAMS is leveraged to transform the proposed hierarchical problem into an MPEC-equivalent problem. Subsequently, we solve the transformed problem by employing the non-linear program with equilibrium constraints (NLPEC) solver available in GAMS. This solution approach offers a robust and effective method for addressing multi-objective non-linear bi-level programming problems with equilibrium constraints, making it applicable to a wide range of challenges within the field. The proposed solution approach was applied to the wood pellet supply chain to investigate the economic and environmental impact of coordination. The results obtained showed that coordination has a positive impact on both economic and environmental performance.

### Comparison of economic and environmental impact under different scenarios

**Fig. 4:**
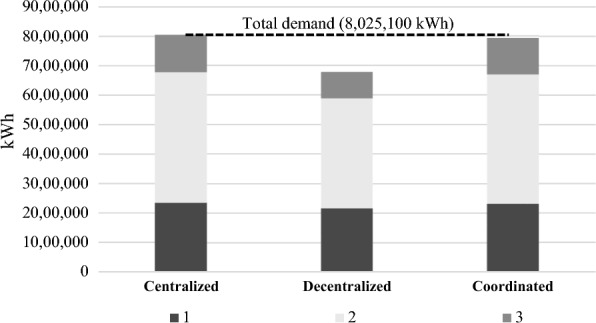
Bioenergy generation in different scenarios

**Fig. 5:**
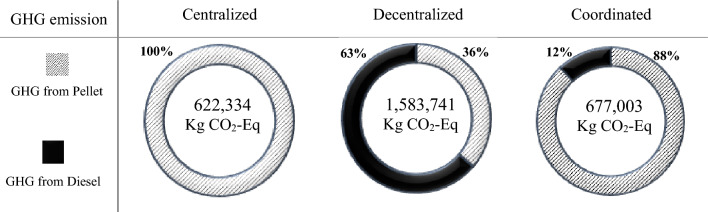
GHG emissions from pellet and diesel through different scenarios to meet the demand

- Economic impact
  The results show that the coordinated approach has a positive impact on the Net Present Value (NPV) of suppliers and wood pellet factories, as shown in Table 4. This improvement reflects the benefits of cooperation and coordination within the supply chain. Conversely, the decrease in NPV for communities in the coordinated scenario indicates that the communities incur extra costs associated with the coordination of the supply chain. However, it is important to note that these extra costs have been partially offset by the increase in bioenergy generation achieved in the coordinated scenario.
  It is noteworthy to mention that the results for the coordinated scenario derived based on the assumption of a revenue-sharing rate initially set at *r* = 10%. As such, to gain a deeper understanding of the impact of changes in this revenue-sharing rate, a sensitivity analysis is presented in Sect. “Sensitivity analysis”. This analysis helps provide insights into identifying revenue-sharing rates for achieving an improved balance between coordination costs and benefits in the bioenergy supply chain.
  In terms of bioenergy generation, the findings presented in this study reveal a substantial disparity in the quantity of wood residues supplied and the corresponding bioenergy generation between a decentralized and a coordinated supply chain. The results clearly demonstrate the impact of coordination on these crucial factors within the supply chain. Table 5 shows that in a decentralized supply chain, the quantity of wood residues provided to the pellet factories (${X}_{ij}^{t}$) is approximately 15% lower than in a coordinated supply chain. This suggests that there may be issues with coordination or communication between the various actors in the supply chain, resulting in a less efficient flow of resources.
  Furthermore, the lower quantity of wood residues in the decentralized supply chain has an impact on the amount of bioenergy generated. The amount of bioenergy generated in the decentralized supply chain is reported as 6,779,300 kWh, which is lower than the coordinated scenario at 7,925,669 kWh (Fig. 4). This suggests that a coordinated supply chain is considerably more effective at maximizing the amount of bioenergy generated from the available resources. In a centralized supply chain, wood pellet can fully satisfy the energy demand in these communities.
- Environmental impact
  The environmental impact analysis of the wood pellet supply chain is crucial to assess the sustainability of bioenergy production. In this study, the CO_2_ equivalent (CO_2_-Eq) emissions of a wood pellet supply chain is assessed throughout its life cycle, including production, transportation, and conversion. The results show that in the centralized scenario, the total CO_2_-Eq emissions from wood pellets were 622,334 kg, which is mainly attributed to the energy consumption for drying, pelletizing, and transportation of wood pellets. In contrast, the decentralized scenario emitted a lower amount of CO_2_-Eq due to lower wood pellet production. However, to meet the remaining energy demand in the communities, diesel was used, which emitted 1,011,741 kg CO_2_-Eq into the air, indicating that diesel combustion contributes significantly to GHG emissions. In the current situation in which diesel is the primary source of energy in Northern communities, the use of 2,431,848 L of diesel results in 6,514,354 kg CO_2_-Eq emissions. This high level of emissions underscores the need for transitioning to sustainable bioenergy sources such as wood pellets to mitigate the negative environmental impact of fossil fuels. Figure 5 demonstrates the proportion of CO_2_ emissions attributed to pellets and diesel in three scenarios.
  The coordinated scenario, which involved quantity discounts and revenue-sharing, significantly reduced the environmental impact to 80,750 kg CO_2_-Eq, which is comparable to the centralized scenario. This outcome indicates that coordination in the wood pellet supply chain can have positive environmental impacts by reducing GHG emissions and contributing to sustainable energy development.
  Overall, the results of the environmental impact analysis emphasize the need for a coordinated approach to the wood pellet supply chain to promote sustainable energy development and mitigate the negative environmental impact of fossil fuels.
  It is worth mentioning that, in the case that we used diesel as the source of energy to satisfy the annual demand in the communities, the CO_2_-Eq emissions were 1,902,069, 3,588,682, and 1,026,602 kg, respectively.

**Table 4:** Players’ NPV in decentralized and coordinated scenarios ($)

Players	Decentralized	Coordinated
Suppliers	322,900	418,480
Wood pellet factories	516,240	598,440
Communities	786,280	708,500

**Table 5:** Total feedstock ordered by pellet factories in various scenarios (kg)

$\sum_{t}{X}_{ij}^{t}$	Centralized		Decentralized		Coordinated	
	*j* = 1	*j* = 2	*j* = 1	*j* = 2	*j* = 1	*j* = 2
*i* = 1	444,100	482,300	366,300	374,000	437,500	428,500
*i* = 2	472,500	524,000	381,700	407,000	448,300	511,000
*i* = 3	461,200	498,800	385,000	374,000	454,600	448,100
Total	2,882,900		2,288,000		2,728,000

### Sensitivity analysis

A sensitivity analysis is carried out to study the impact of the key coordination parameter, revenue-sharing rate (*r*) on the performance of the coordinated supply chain (i.e., players’ NPV and bioenergy generation). In doing so, this parameter is changed while keeping all other parameters constant. Observing the outcomes help assess how different revenue-sharing rates might impact the NPV of the involved parties providing insights into the best rates for achieving an improved balanced between coordination costs and benefits in the bioenergy supply chain.

**Fig. 6:**
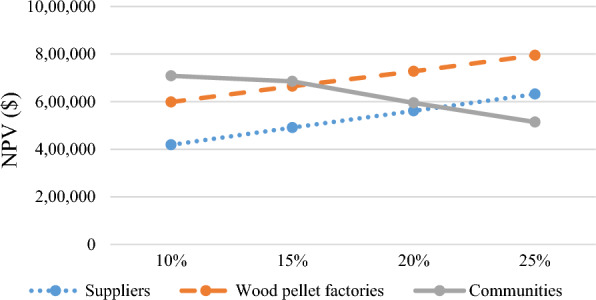
Impact of revenue-sharing rate (*r*) on the NPV of supply chain players ($)

**Fig. 7:**
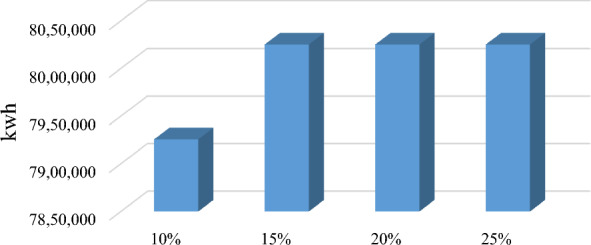
Impact of revenue-sharing rate (*r*) on bioenergy generation (kwh)

Figures 6 and 7 show that the threshold value of the revenue-sharing rate approximately remains at 15%. In other words, an excessively high revenue-sharing rate does not contribute to improving the coordination of the supply chain. This observation is crucial, as it highlights the critical point where increasing the rate further does not improve coordination benefits. The rationale behind this phenomenon is that when the revenue-sharing rate surpasses 15%, the communities' NPV will decrease. This decline occurs even though the bioenergy generation remains constant at its maximum capacity, satisfying 100% of the communities’ demand. Therefore, the reduction in communities’ NPV does not justify sharing the revenues at a higher rate, emphasizing the importance of finding the right balance between revenue-sharing and the financial well-being of the communities.

This finding underscores the significance of not only identifying the best performing revenue-sharing rate but also considering the implications on different stakeholders, especially the small communities in the off-grid region in the absence of efficient energy storage systems. In summary, it highlights the need for a nuanced approach to revenue-sharing that maximizes the benefits of coordination while safeguarding the economic interests of all parties involved.

## Conclusions

This study highlighted the importance of coordination in the wood pellet supply chain for both economic and environmental factors. It suggested a coordinated approach that includes quantity discounts and revenue-sharing to address the issue of conflicting interests among different participants in the supply chain. By focusing on the adoption of biomass as an alternative source for electricity generation in Quebec northern communities, the study acknowledged the energy security concerns faced by isolated regions heavily reliant on diesel fuel (Canada’s Energy Future, 2016). This approach not only demonstrated the potential for improved economic performance but also showcased a substantial reduction in environmental impact.

However, it is important to recognize that the findings of this study are specific to the case study conducted in three Quebec northern communities. Therefore, the generalizability of the results to other regions or contexts may be limited, influenced by transportation options and the availability of biomass residues. Furthermore, the assumptions and parameters used in the coordination model, such as quantity discounts and revenue-sharing mechanisms, may not fully capture the complexities of real-world supply chain dynamics. Future research should include an in-depth analysis of potential risks and barriers associated with the adoption of biomass as an alternative energy source in isolated communities. This includes the availability of transport means for biomass, access to precise energy demand predictions, as well as assessment of biomass sourcing risks (Esmaeili et al., 2023). Moreover, it is essential to consider potential unintended consequences or trade-offs that could arise from policies promoting bioenergy usage and reducing diesel dependence. Further investigations should explore the impact of various factors on the wood pellet supply chain, including government policies and regulations (Liu et al., 2022). This analysis should consider economic and environmental trade-offs, such as investment costs, job creation, and carbon emission across biomass supply chain activities (Sadaghiani et al., 2023).

To advance the field, future studies should delve into quantifying the economic performance improvements resulting from the proposed coordinated approach and measuring the achieved reduction in environmental impact. In addition, the scalability of the model should be assessed, exploring its implications for large-scale implementation within the wood pellet industry. In addition, the incorporation of uncertainties and dynamism in biomass supply chain coordination models (Khoddami et al., 2021). Addressing these research directions could further contribute to improving the performance of coordination schemes in wood pellet supply chains enhancing their adoption as a sustainable energy source.

## Data Availability

Data used in this study are available upon request from the corresponding author.

## Appendix 1

Cost of biomass transportation from supplier ‘*i*’ to hub ‘*k*’ ($/kg)

**Table:**

		Pellet factories	
		1	2
Suppliers	1	0.012	0.015
	2	0.011	0.016
	3	0.012	0.015
	4	0.014	0.012
	5	0.015	0.011
	6	0.016	0.010

## Appendix 2

Capacity of suppliers (Si) (kg), biomass price of suppliers with and without discount ($${P}_{i}^{l}, {P}_{i}^{u}$$) ($/kg)

**Table:**

	***S***_***i***_	${{\varvec{P}}}_{{\varvec{i}}}^{{\varvec{l}}}$	${{\varvec{P}}}_{{\varvec{i}}}^{{\varvec{u}}}$
1	33,300	0.168	0.205
2	34,000	0.170	0.210
3	34,700	0.175	0.200
4	37,000	0.190	0.215
5	35,000	0.190	0.220
6	34,000	0.185	0.220

## Appendix 3

Wood pellet ordering cost from pellet factory ‘*j*’ to community ‘*k*’ ($${P}_{jk}^{l}, {P}_{jk}^{u}$$) ($/kg)

**Table:**

	Communities			
		1	2	3
Wood pellet factory	1	(0.235, 0.362)	(0.235, 0.362)	(0.235, 0.362)
	2	(0.266, 0.409)	(0.266, 0.409)	(0.266, 0.409)

## Appendix 4

Demand in community ‘*k*’ at time ‘*t*’ (12 months) (kWh)

**Table:**

	1	2	3
1	186,300.000	351,500.000	100,500.000
2	171,900.000	324,400.000	92,800.000
3	171,000.000	322,600.000	92,300.000
4	180,900.000	341,200.000	97,600.000
5	179,700.000	339,100.000	97,000.000
6	168,700.000	318,300.000	91,100.000
7	178,700.000	337,100.000	96,400.000
8	194,800.000	367,500.000	105,100.000
9	216,800.000	409,000.000	117,000.000
10	246,900.000	465,900.000	133,300.000
11	226,500.000	427,400.000	122,300.000
12	219,900.000	414,900.000	118,700.000

## List of Symbols

*i*: Set of suppliers
*j*: Set of pellet factories
*k*: Set of energy convertor facilities (Communities)
*t*: Time periods
*r*: The revenue-sharing rate
${z}_{k}^{t}$: Electricity generation from biomass in community *k* at the time *t*
${Z}_{k}$: Capacity of electricity generation in community *k*
${LD}_{k}$: Electricity generation cost from diesel
${f}_{c}$: The conversion rate of biomass
${P}_{ij}^{t}$: Feedstock price of supplier *i* for wood pellet factory *j*
${P}_{jk}^{t}$: Wood pellet price of factory *j* for community *k*
$${C}_{con}$$: Conversion cost
${D}_{k}^{t}$: Demand in community *k* at time *t*
${X}_{ij}^{t}$: Quantity of feedstock delivered from supplier *i* to factory *j* at the time *t*
${X}_{jk}^{t}$: Quantity of wood pellet delivered from factory *j* to community *k* at the time *t*
${CF}_{S}$: The carbon footprint of the upstream process
${CF}_{P}$: The carbon footprint of the midstream process
${CF}_{C}$: The carbon footprint of the downstream process
$${C}_{ha}$$: Harvesting cost
$${CF}_{de}$$: Debarking cost
${CF}_{sa}$: Head sawing cost
${CF}_{tp(ij)}$: Transportation cost of feedstock from supplier *i* to pellet factory *j*
$${CF}_{dr}$$: Drying cost
$${CF}_{mi}$$: Milling cost
$${CF}_{pe}$$: Pelletizing cost
$${CF}_{co}$$: Cooling cost
${CF}_{tc}$: Transportation cost to the location of communities
$${CF}_{con}$$: Conversion cost
${CF}_{tf}$: Transmission cost to the place of final customer
${S}_{i}$: The capacity of supplier ‘*i*’
${S}_{j}$: The capacity of pellet factory ‘*j*’
${NPV}_{C}$: Net present value of communities
${NPV}_{S}$: Net present value of suppliers
${NPV}_{P}$: Net present value of pellet factories

## Abbreviations

GHG: greenhouse gas
LCA: Life cycle analysis
LCO: Life cycle optimization
NPV: Net present value
TEA: Techno-economic assessment
